# An Experimental Murine Model to Assess Biofilm Persistence on Commercial Breast Implant Surfaces

**DOI:** 10.3390/microorganisms10102004

**Published:** 2022-10-11

**Authors:** Francisco Carmona-Torre, Leire Fernández-Ciriza, Carlos Berniz, Cristina Gomez-Martinez de Lecea, Ana Ramos, Bernardo Hontanilla, Jose L. del Pozo

**Affiliations:** 1 Infectious Diseases Division, Clínica Universidad de Navarra, 31008 Pamplona, Spain; 2 IdiSNA, Navarra Institute for Health Research, 31008 Pamplona, Spain; 3 Microbiology Department, Clínica Universidad de Navarra, 31008 Pamplona, Spain; 4 Department of Plastic Surgery, Clínica Universidad de Navarra, 31008 Pamplona, Spain

**Keywords:** breast, implant, biofilm, pathogenesis, staphylococcal, Wistar rat, texturized, capsular contracture, Mentor, McGhan, Allergan

## Abstract

Capsular contracture is the most frequently associated complication following breast implant placement. Biofilm formation on the surface of such implants could significantly influence the pathogenesis of this complication. The objective of this study was to design an experimental model of breast implant infection that allowed us to compare the in vivo *S. epidermidis* ability to form and perpetuate biofilms on commonly used types of breast implants (i.e., macrotexturized, microtexturized, and smooth). A biofilm forming *S. epidermidis* strain (ATCC 35984) was used for all experiments. Three different implant surface types were tested: McGhan BIOCELL^®^ (i.e., macrotexturized); Mentor Siltex^®^ (i.e., microtexturized); and Allergan Natrelle Smooth^®^ (i.e., smooth). Two different infection scenarios were simulated. The ability to form biofilm on capsules and implants over time was evaluated by quantitative post-sonication culture of implants and capsules biopsies. This experimental model allows the generation of a subclinical staphylococcal infection associated with a breast implant placed in the subcutaneous tissue of Wistar rats. The probability of generating an infection was different according to the type of implant studied and to the time from implantation to implant removal. Infection was achieved in 88.9% of macrotextured implants (i.e., McGhan), 37.0% of microtexturized implants (i.e., Mentor), and 18.5% of smooth implants (i.e., Allergan Smooth) in the short-term (*p* < 0.001). Infection was achieved in 47.2% of macrotextured implants, 2.8% of microtexturized implants, and 2.8% of smooth implants (i.e., Allergan Smooth) in the long-term (*p* < 0.001). There was a clear positive correlation between biofilm formation on any type of implant and capsule colonization/infection. Uniformly, the capsules formed around the macro- or microtexturized implants were consistently macroscopically thicker than those formed around the smooth implants regardless of the time at which they were removed (i.e., 1–2 weeks or 3–5 weeks). We have shown that there is a difference in the ability of *S epidermidis* to develop in vivo biofilms on macrotextured, microtextured, and smooth implants. Smooth implants clearly thwart bacterial adherence and, consequently, biofilm formation and persistence are hindered.

## 1. Background

Silicone gel breast implants are the most often used devices for aesthetic and reconstructive breast surgery. Most manufacturers have modified the composition of the silicone gel, as well as the degree of texturization of the elastomers, providing a wide variety of implant models that can be individualized according to the type of patient in whom they are going to be placed [1,2].

Capsular contracture is the most common complication following breast implant placement, appearing in up to 20% of cases [3]. Several studies have related biofilm formation on the surface of the implant with capsular contracture development [4,5,6]. Some in vitro studies show that the adherence capacity of bacteria to texturized implants is higher than that to non-texturized implants, and that there is an association between biofilm formation on implants and the host response leading to a capsular contracture [7]. In fact, several studies have related in vivo biofilm formation on the surface of the implant with capsular contracture development [4,5,6]. However, clinical studies attempting to correlate the use of texturized implants and the possible increase in the incidence of capsular contracture are extremely controversial [8].

There is currently a lack of experimental models to study the association of capsule contracture and the host response. The objective of this study was to design an experimental model of breast implant infection that allows us to compare the in vivo ability of the *S. epidermidis* to form and perpetuate biofilms on commonly used types of breast implants (i.e., macrotexturized, microtexturized, and smooth).

## 2. Material and Methods

### 2.1. Bacterial Strain

A biofilm forming *S. epidermidis* strain ATCC 35984 was used for all experiments.

#### 2.1.1. Implant Surfaces

Three different implant surface types were tested: Allergan Natrelle Smooth^®^ (i.e., smooth); Mentor Siltex^®^ (i.e., microtexturized); and McGhan BIOCELL^®^ (i.e., macrotexturized). Implants were prepared by cutting a 1 cm^2^ square section of the implant shell from the whole implant and scraping away any residual silicone from the inner surface to avoid possible artifacts arising from passage of the silicone mass into the peri-implant capsule. The implant pieces were sterilized under dry heat conditions at 120 °C for 1 h.

#### 2.1.2. Implant Colonization

Pieces of implant samples were attached to the polypropylene holders of a CBR 90 Standard CDC Biofilm Reactor (Biosurfaces technologies corporation, Bozeman, MO, USA). Then, one ml of brain heart infusion (BHI) containing 10^5^ cells/mL of *S. epidermidis* was incubated for 30 min to obtain log phase growth, added to the Biofilm Reactor, and incubated in continuous shaking at 37 °C for up to 48 h. Implant samples were removed at 48 h and washed three times in 10 mL of phosphate-buffered saline prior to implantation. To confirm the absence of contamination during processing and the presence of an adequate bacterial inoculum, two implants of each type (i.e., smooth, microtexturized, and macrotexturized) were randomly processed at the time of implant extraction for each experiment. Those control implants were placed in 1 mL of BHI, vortexed for 30 s, and subjected to sonication for 5 min followed by other 30 s of vortexing. Quantitative numbers of bacteria attached to the implant were determined by serial 10-fold dilutions and standard plate culture.

### 2.2. Experimental Model

The experimental protocol was approved by the Ethics Committee of University of Navarra. Every aspect of animal housing and experimentation was considered to minimize suffering and treatment was performed according to the European Commission. Sixty-three 9-week-old Wistar rats were operated on with subcutaneous implantation of three colonized implants per animal. The median weight was 297 (SD 46.6) grams (96% IC 288.7–305.3 g). All surgeries were performed in a laminar air flow cabinet under standard operating theatre conditions. In all experiments, the skin was shaved and washed with 2% chlorhexidine plus alcohol. The animals were placed on sterile drapes for surgery. In all operations, the rats were given general inhaled sedation (i.e., inhaled isoflurane at a concentration of 2%) and, if necessary, carprofen as postoperative pain treatment. At the end of each experiment, the rats were killed with cervical dislocation after sedation.

Briefly, three 1 cm-long incisions were made in the midline at the proximal part of the back and on each side of the midline at the distal part of the back (right and left), and three independent subcutaneous cavities were made with scissors. In animals that acted as negative in vivo controls, sterile implants were placed directly into every subcutaneous pocket. In the rest of the animals, the respective colonized implants were implanted. Surgical staples for skin closure were used. All animals were operated on separately, with new dressing and sterile instruments for each animal. The animals were housed in a 12 h night–day cycle environment and were fed ad libitum. The extent of infection was evaluated by clinical observation of the animals.

**Figure 1:**
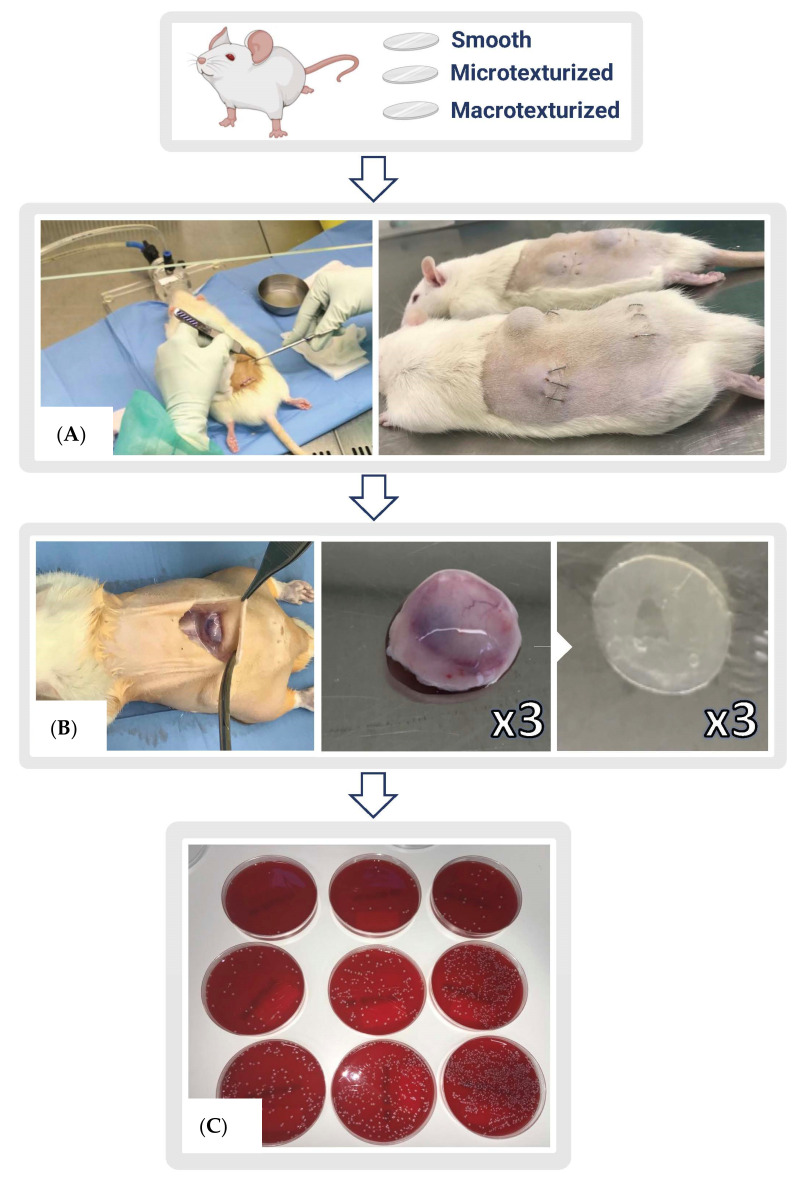
(**A**) Colonized implants were embedded in a laminar air flow cabinet under standard operating theatre conditions. Three 1 cm-long incisions were made, implants were placed in the subcutaneous layer, and surgical staples were used for skin closure. (**B**) Extraction of the capsules containing the implants was performed under sterile conditions. (**C**) Quantitative post-sonication culture of implants and capsule biopsies was performed (in the serial dilution plates we can observe the differences among the three implant types (i.e., smooth, microtextured, and macrotextured). (Completed using the BioRender.com tool).

**Figure 2:**
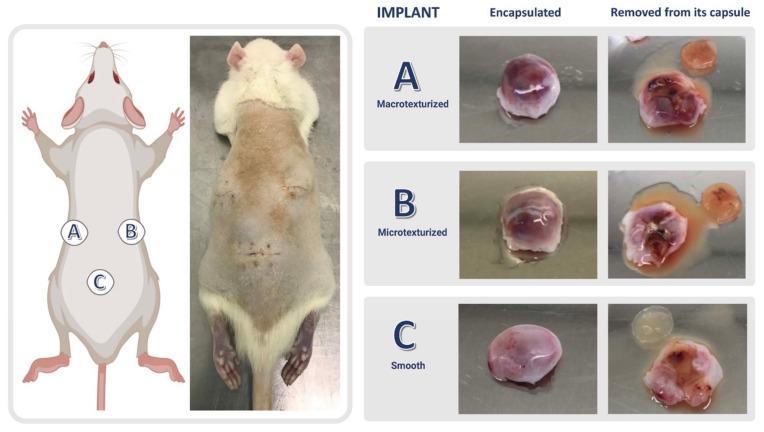
Location of implants in the Wistar rat ((**A**) macrotexturized, (**B**) microtexturized, (**C**) smooth). Macroscopic appearance of the capsules containing each type of implant. (Prepared using the BioRender.com tool).

Two different infection scenarios were simulated: (i) short-term infection (i.e., acute infection occurring less than 14 days from implantation), and (ii) long-term infection (i.e., subacute-chronic infection occurring beyond 21 days post-implantation). Accordingly, implants were removed for culture between days 7 and 14 or between days 21 and 35. Control negative implants were extracted at all time points. In the same way, the respective capsules formed around the implants were extracted under sterile conditions at the same time points. Both elements were weighed and processed separately. Quantitative cultures of the implants and capsules were performed on Columbia agar +5% sheep blood after vortexing for 30 s, sonication for 5 min, and a further 30 s of vortexing in 1 mL of BHI. We confirmed the absence of contamination by MALDI-TOF MS identification (VITEK-MS) and automated antibiogram of the isolated colonies (VITEK-2). The extent of infection was evaluated by clinical observation of the animals. The degree of biofilm formation on capsules and implants was evaluated by quantitative post-sonication culture of implants and capsule biopsies following the aforementioned protocol (Figure 1 and Figure 2).

### 2.3. Statistical Analysis

Qualitative variables are be expressed as mean + standard deviation or median accompanied by the interquartile range (IQR), and qualitative variables are expressed as numbers and percentages. Normality of distributions was verified by means of the Shapiro–Wilk test. For the comparison of bacterial counts between the different types of implants, in the short- and the long-term infection models, we confirmed the absence of fit to normality despite a log transformation. Thus, we employed the Friedman test with the Sidak and Holm post hoc test. Categorical variables were evaluated using a chi-square test or Fisher’s exact test when appropriate. Correlations between continuous variables were evaluated by calculating Pearson’s correlation coefficient or, when appropriate, Spearman’s correlation coefficient. All *p*-values presented are two-tailed. Prism 9 software (GraphPad Software, San Diego, CA, USA) and STATA13.0 were used for statistical analysis.

## 3. Results

The experimental model that we developed allows the generation of a subclinical staphylococcal infection associated with a breast implant placed in the subcutaneous cellular tissue of Wistar rats. No animal developed clinical signs of disseminated infection or required an earlier endpoint. No implant contamination occurred (i.e., defined as an implant culture isolation of a microorganism different from the studied strain). Capsule contamination (i.e., defined as a capsule culture isolation of a microorganism different from the studied strain) occurred in three animals either during placement or during subsequent removal and processing in three animals (i.e., three capsules, one of each type in each animal, with *Staphylococcus aureus*). These three capsules were excluded from the final analysis of the study. Bacterial counts (median and IQR) after logarithmic transformation for infected prosthesis prior to implantation were: smooth 18.30 (18.27–18.30); microtexturized 17.88 (17.55–18.65); and macrotexturized 19.01 (19.0–19.02). This model made it possible to generate an implant-associated infection in a total of up to almost 50% (i.e., 48.15% in implants colonized in the short term) of the animals that underwent surgery. The probability of generating an infection was different according to the type of implant studied and to the time from implantation to implant removal (Table 1).

**Figure 3:**
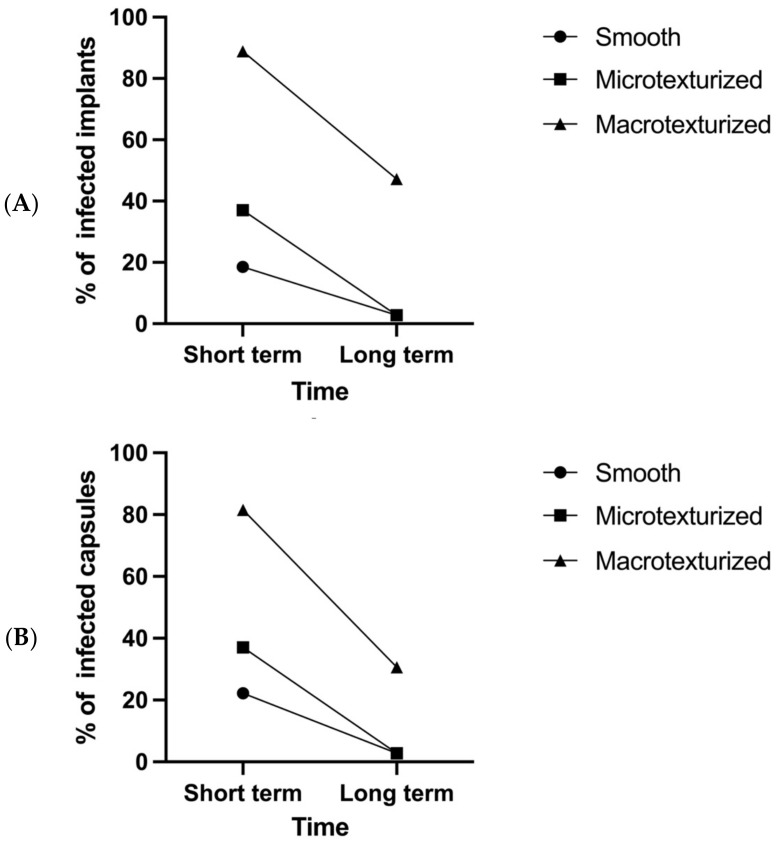
(**A**). Implant. Evolution of implant related infection over time. (**B**). Capsules. Evolution of capsule infections in the model over time.

Figure 3 shows the percentage of implants colonized in the short term (i.e., 1–2 weeks) and in the long term (i.e., 3–5 weeks) according to implant surface topography. As can be seen, macrotexturized implants show the highest percentage of colonized implants. In addition, a decrease in the percentage of colonized implants was observed over time, regardless of the type of implant examined. Infection was achieved in 88.9% of macrotextured implants (i.e., McGhan), 37.0% of microtexturized implants (i.e., Mentor), and 18.5% of smooth implants (i.e., Allergan Smooth) in the short term (*p* < 0.001). Infection was achieved in 47.2% of macrotextured implants (i.e., McGhan), 2.8% of microtexturized implants (i.e., Mentor), and 2.8% of smooth implants (i.e., Allergan Smooth) in the long term (*p* < 0.001).

**Figure 4:**
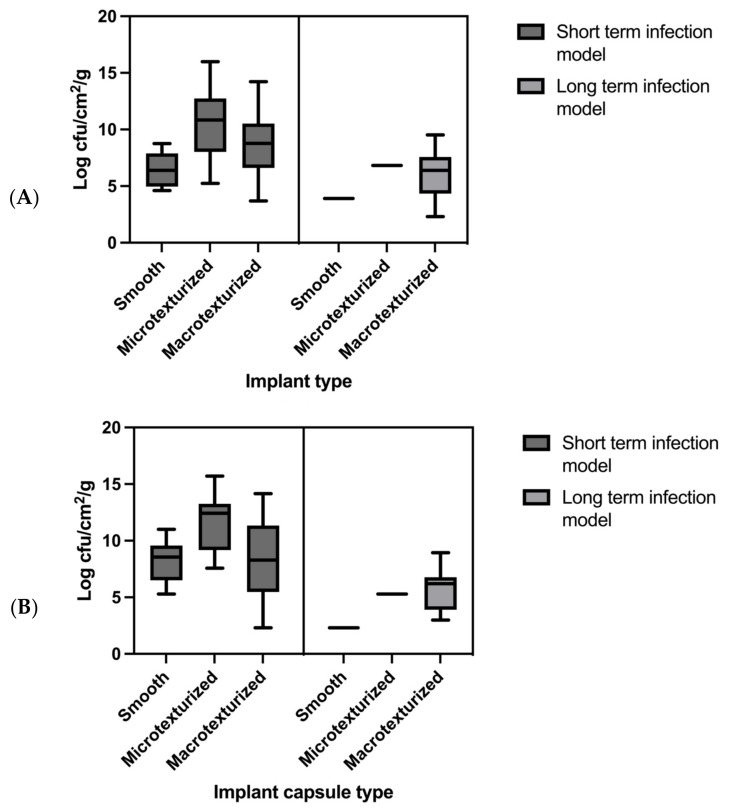
(**A**). Implant bacterial counts in short and long-term infection. (**B**). Capsule bacterial counts in short and long-term infection.

The median and IQR of the logarithmic transformation of bacterial counts for the different types of implants (i.e., Natrelle Smooth, Mentor, and McGhan) in the short-term and long-term infection models are shown in Table 2 and Figure 4. Statistically significant differences were demonstrated when we compared the ability to generate a stable infection on the three types of implants in the short-term model (*p* < 0.001). We used Friedman’s test (paired samples) for comparison of *S. epidermidis* bacterial counts on the three different implant types. A comparative analysis by group shows statistically significant differences between macrotexturized and smooth implants (*p* < 0.001) and between macrotexturized and microtexturized implants (*p* = 0.013). We also observed a trend towards statistical significance when comparing microtexturized and smooth implants (*p* = 0.065). Statistically significant differences were demonstrated when we compared the ability to generate a stable infection on the three types of implants in the long-term model (*p* = 0.005). We used Friedman’s test (paired samples) for comparison of *S. epidermidis* bacterial counts on the three different implant types. A comparative analysis by group shows statistically significant differences between macrotexturized and smooth implants (*p* < 0.001) and between macrotexturized and microtexturized implants (*p* = 0.002). We did not observe statistical significance differences when comparing microtexturized and smooth implants (Table 3).

**Figure 5:**
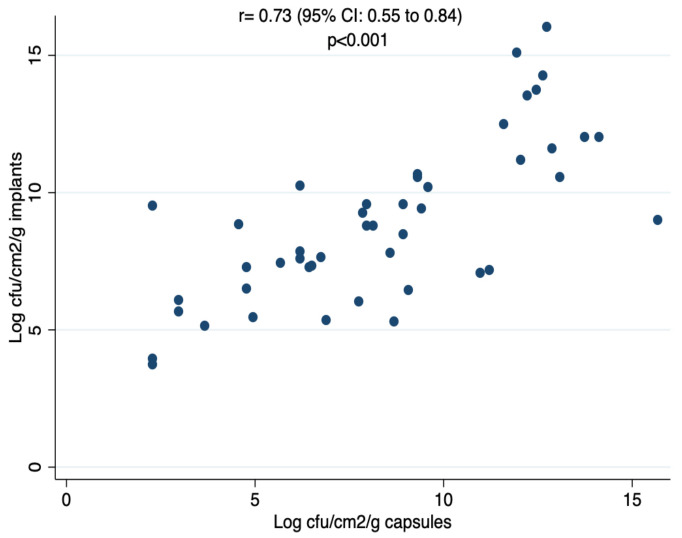
Scatter plot and correlation coefficients for global implant and capsule log cfu/cm^2^/g counts.

There was a clear positive correlation between biofilm formation on any type of implant and capsule infection [r = 0.73 (95% CI: 0.55 to 0.84)] (Figure 5). The capsules uniformly formed around the macro- or microtexturized implants were consistently macroscopically thicker than those formed around the smooth implants regardless of the time at which they were removed (i.e., 1–2 weeks or 3–5 weeks). As an approximation, a summary of capsule weights at different stages of infection is shown in Table 4. Histopathological examination of the capsules was outside of the scope of the present study.

## 4. Discussion

In this study we present a new in vivo experimental model for the study of breast implant-associated infection. This model allows in vivo evaluation of the different biofilm-forming capacities over time of a strain of *S. epidermidis* on several types of breast implants that are frequently used in clinical practice. Basically, it allows analysis of the capacity to perpetuate the infection over time according to the topographical characteristics of the implant surface.

We demonstrated that an implant-associated infection is more likely to be generated and maintained in the case of macro- or microtextured implants when compared with smooth ones. We found significant differences between the percentages of implants that remain colonized in the short and long term. It seems that it is more difficult to generate an infection associated with a smooth implant compared to a textured one (i.e., Mentor, McGhan). Furthermore, when we analyzed the bacterial count attached to the surface of the implants, we also found significant differences among the three different types of implants. The counts were highest for microtextured implants, then for macrotextured implants, and the lowest counts were observed for smooth implants. Statistically significant differences between the bacteria adhered to smooth and macrotextured implants remained constant in the short- and long-term models, as do those observed between macrotextured and microtextured implants, adding robustness to our findings. However, the impact of these findings on capsule thickness does not apparently follow a direct relationship, as was reported in other studies [9,10]. The means capsule weight for each type of implant could support this lack of relation in our series. In other words, the combination of the implant roughness [9], the bacterial count attached to the implant, and the host response [10] could be the probable conditioning factors of the development of capsular contracture.

In recent years, many in vitro models have been described for the study of capsular contracture associated with infection. However, the main limitation of in vitro models is that they do not consider the interaction between the host, the bacteria, and the implant. To the best of our knowledge, no experimental breast implant infection model has been standardized to date. The experimental model we designed has the following characteristics: (a) The surgical procedure is technically easy to perform. Surgeries of the animals can be performed by personnel not necessarily possessing complete surgical skills. (b) The model allows several different implants to be used in the same animal. (c) The established infection has the characteristics of a chronic local infection. (d) The risk of bacterial contamination from the fur and skin of the animal is negligible.

The development of this model allows the generation of acute and chronic infections (i.e., infections developed 3–5 weeks after surgery) that can be useful for studying the genesis of capsular contracture. Moreover, this model allows the study of the formation of biofilms on the implants, in addition to the microbiological and histopathological analysis of the peri-implant capsules. In this sense, the possibility of generating a capsular contracture model may allow us to evaluate the efficacy of different antibiotic treatments to prevent this complication. This would be especially applicable to infectious models based on macrotexturized implants, since the proportion of infections in this group is very stable (88.9% in the short-term model and 47.2% in the long-term model), rising to 72.2% in the latter group if we consider the positive cultures obtained after prolonged incubation of the implants in enrichment media, in comparison with 8.3% for microtexturized and 5,5% for smooth implants.In all cases, we confirmed antibiogram phenotypical concordance with the experimental *S. epidermidis* strain (ATCC 35984). Furthermore, experiments based on smooth and microtextured implants are possible but will require an increase in sample size to avoid loss of statistical power. This model also allows the placement of different types of implants in the same animal. This is undoubtedly a great advantage when homogenizing the results because it eliminates the variability that may exist between animals.

Several clinical and laboratory studies have shown that the presence of bacteria on the surface of breast implants contributes significantly to the development of capsular contracture related to the implant [11,12]. Some in vitro work has shown that the topography of the implant surface influences the adhesion of eukaryotic cells and microorganisms [7,13]. Texturization of the outer surface of breast implants was first used in 1968 with the “natural Y” implant, which incorporated a 1.2 to 2 mm polyurethane coating on its outer surface. This surface design was able to prevent the organized alignment of myofibroblasts, thus reducing the risk of capsular contracture development [14]. Mentor embraced the idea and released the first textured breast implant, Siltex, in 1987. McGhan corporation (Santa Barbara, CA, USA) followed shortly after with its Biocell textured surface. The benefits of using textured implants to reduce capsular contracture are controversial based on data published in the literature [15]. Many published papers lack adequate description of implant type, surgical technique, and evaluation of results. Overall, it appears that bacterial adherence to textured implants is higher than to other types of implants in vitro [16]. This result agrees with ours, where we observed that the percentage of implants colonized in both the short and long term, and the number of bacteria capable of perpetuating on an implant, are lower in the case of smooth implants compared with textured implants. In the same way, textured implants demonstrated a three-fold higher infection rate over comparable smooth implants in a prospective series [17]. However, the role played by the texture of the breast implant in the development of a biofilm on its surface is not entirely clear. Scherml et al. found no difference when comparing adherence to smooth or textured implants [18]. On the contrary, Jacombs et al. described a higher biofilm development on textured compared to smooth implants in vitro after 24 h of incubation in vitro [16]. It seems reasonable to hypothesize that implant topography may affect the development of biofilms on the implant.

A correlation between a higher degree of bacterial adherence and host response in vivo was demonstrated, suggesting that biofilms formed on the surface of implants trigger an inflammatory host response [19]. Several studies have proposed that the development of biofilms on the surface of breast implants could be the cause of the pathogenesis of the development of a chronic inflammatory response leading to the development of capsular contracture [4,5,12,18,20,21,22]. In this regard, it is striking that the biofilms formed on texturized implants persist for at least 5 weeks after the challenge. Similarly, the cultures of peri-implant biopsies are positive for a longer period in the case of texturized implants. It is likely that the persistence of *S. epidermidis* in the capsules surrounding rough implants may enable progression to capsular contracture due to the presence of a sustained antigenic stimulus.

We showed that there is a difference in the ability of *S epidermidis* to develop in vivo biofilms on macrotextured, microtextured, and smooth implants. Smooth implants clearly thwart bacterial adherence and, consequently, biofilm formation is hindered. Moreover, as can be seen in the figures, the persistence of biofilms over time is clearly lower in the case of smooth implants. Therefore, capsule infection may occur less frequently when smooth implants are used. This could explain the lower rate of capsular contracture observed in some series of patients with smooth implants [16]. The capsule formed around macrotextured implants was much more prominent and thicker than the one formed around the smooth implants [9]. This fact could be related to the incipient development of a capsular contracture. However, it would be very interesting to corroborate this fact by performing histopathological studies on the capsules formed. The persistence of capsular biofilms is only indirect evidence that a subclinical infection could be behind the development of capsular contracture. It would then be reasonable to propose the use of antibiotic treatments to reduce the risk of capsular contracture.

As mentioned above, the texture of the implant surface enables the biointegration with the host tissue, but also the possibility of biofilm formation on the implant. It is possible that other factors, in addition to the texture of the implant, such as the antibiotic irrigation of the pocket, the prophylactic use of antibiotics, the sterile technique when placing the implant, or the anatomical location of the pocket, have an important influence on the formation of biofilms on the implant [23].

The main limitation of this study is that only one bacterial species (i.e., *S. epidermidis*) was studied. The study of other microorganisms that have also been related to the formation of capsular contracture, such as *Cutibacterium acnes*, would be of great interest. In addition, biofilm quantification studies could have been complemented with imaging techniques to make a qualitative assessment of the presence of biofilms.

The design and development of this animal model has allowed us to better understand the potential pathogenesis of breast implant-related infections. It has also allowed us to analyze the differences in the capacity of adherence to different types of breast implant. This fact can have an important clinical repercussion since the formation of biofilms on the surface of the implants may have a direct impact on the development of capsular contracture. This model may have future utility for the evaluation of the efficacy of preventive or eradicating treatments of biofilms in vivo and their impact on the genesis of capsular contracture.

## Figures and Tables

**Table 1:** Implant-associated infection rate according to implant type.

Implant Type	Implant		Capsule	
	Short-Term	Long-Term	Short-Term	Long-Term
Smooth, *n* (%)	5 (18.5)	1 (2.8)	6 (22.2)	1 (2.8)
Microtexturized, *n* (%)	10 (37)	1 (2.8)	10 (37)	1 (2.8)
Macrotexturized, *n* (%)	24 (88.9)	17 (47.2)	22 (81.5)	11 (30.6)
Total positive cultures	39 (48.1)	19 (17.6)	38 (46.9)	13 (12.1)
Total samples, *n* (%)	81 (100)	108 (100)	81(100)	108 (100)
Total animals, *n*	27	36	27	36

**Table 2:** Summary of median and interquartile range (IQR) of the logarithmic transformation of bacterial counts (cfu/cm^2^/g) for every implant and capsule type.

Infection Stage	Implant	Capsule
Short term		
Smooth, median (IQR)	6.4 (5.3–7)	8.6 (6.9–9.1)
Microtexturized, median (IQR)	10.8 (8.9–12)	12.4 (9.3–13.1)
Macrotexturized, median (IQR)	8.8 (6.8–10.4)	8.3 (5.7–11.2)
Long term		
Smooth, median (IQR)	3.9 (3.9–3.9)	2.3 (2.3–2.3)
Microtexturized, median (IQR)	6.8 (6.8–6.8)	5.3 (5.3–5.3)
Macrotexturized, median (IQR)	6.4 (4.6–7.5)	6.2 (3.9–6.8)

**Table 3:** Adjustment of *p*-values for multiple comparisons of each implant by Holm test and SIDAK post hoc test.

Infection Stage	Implants			
	Friedman Test	*p*-Value Unadjusted for Multiple Comparisons	SIDAK Post-Hoc Test	HOLM Post-Hoc Test
Short-term				
Microtexturized vs. Smooth (*p*)	<0.001	0.065	0.184	0.065
Macrotexturized vs. Smooth (*p*)		<0.001	<0.001	<0.001
Macrotexturized vs. Microtexturized (*p*)		0.007	0.02	0.013
Long-term				
Microtexturized vs. Smooth (*p*)	0.005	1.0	1.0	1.0
Macrotexturized vs. Smooth (*p*)		<0.001	<0.001	<0.001
Macrotexturized vs. Microtexturized (*p*)		<0.001	0.002	0.001

**Table 4:** Median and interquartile range (IQR) of capsule weights without implant in each infection stage.

Implant Types	Infection Stage	Capsule Weight
Smooth, median (IQR)	Short term (*n* = 27)	0.5162 (0.2309–0.643)
	Long term (*n* = 36)	0.2341 (0.206–0.3176)
Microtexturized, median (IQR)	Short term (*n* = 27)	0.5569 (0.4367–0.9367)
	Long term (*n* = 36)	0.3135 (0.2524–0.4039)
Macrotexturized, median (IQR)	Short term (*n* = 27)	0.7582 (0.4323–0.9056
	Long term (*n* = 36)	0.31315 (0.25525–0.41565)

## Data Availability

The data presented in this study are available on request from the corresponding author. The data are not publicly available because they are part of work pending publication.
